# Recurrent Mural Thrombosis of the Ascending Aorta in a Patient with Antiphospholipid Syndrome

**DOI:** 10.3400/avd.cr.21-00138

**Published:** 2022-03-25

**Authors:** Ryohei Otsuka, Shunei Saito, Toshikuni Yamamoto, Tsukasa Ohno, Akio Koyama, Hirofumi Morimae, Masahiro Matsushita, Kaori Yokota, Ken Miyahara, Akio Matsuura

**Affiliations:** 1 Division of Cardiovascular Surgery, Ichinomiya Municipal Hospital, Ichinomiya, Aichi, Japan; 2 Division of Vascular Surgery, Ichinomiya Municipal Hospital, Ichinomiya, Aichi, Japan; 3 Division of Rheumatology and Collagen Diseases, Ichinomiya Municipal Hospital, Ichinomiya, Aichi, Japan

**Keywords:** aortic operation, thrombosis, antiphospholipid syndrome

## Abstract

A 38-year-old man presented with embolic occlusion of the brachial artery. As per his computed tomography results, a pedunculated mass in the proximal ascending aorta was detected. Since discrimination between a thrombus and a tumor was deemed difficult, the patient underwent replacement of the ascending aorta. Histopathology revealed the mass to be a thrombus. The diagnosis of antiphospholipid syndrome was then confirmed postoperatively. Six months post-surgery, a new thrombus was detected in the vascular prosthesis. The thrombus resolved after treatment with edoxaban and aspirin. To the best of our knowledge, this is the first report on graft thrombosis in antiphospholipid syndrome, highlighting the importance of seamless anticoagulation therapy.

## Introduction

Although arterial and venous thromboses at various sites are identified as manifestations of antiphospholipid syndrome (APS), mural thrombosis of the aorta is rare. Moreover, distinguishing a mural thrombus from an aortic tumor remains to be a challenge. Herein, we report a case of recurrent mural thrombosis of the ascending aorta in a patient diagnosed with APS after the replacement of the ascending aorta.

## Case Report

**Figure 1:**
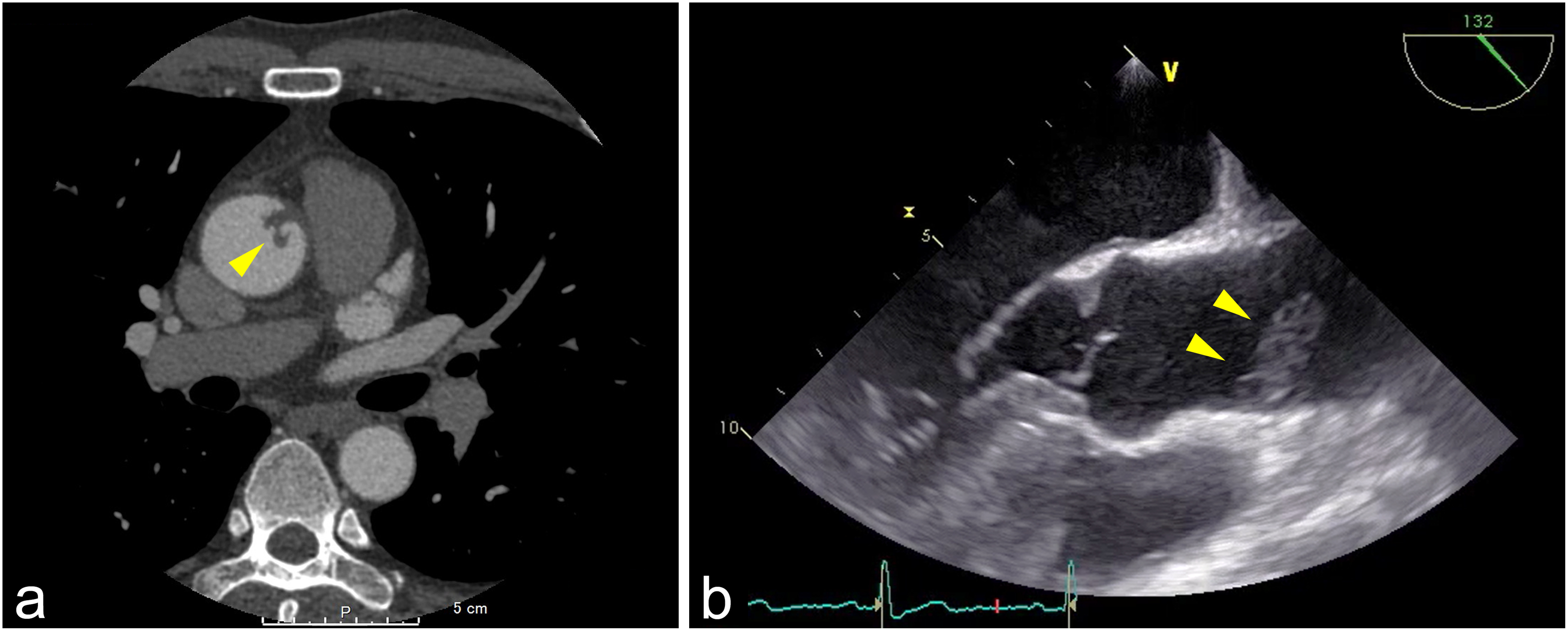
Fig. 1 (**a**) Computed tomography scan and (**b**) transesophageal echocardiogram showing a mass (arrowheads).

A 38-year-old man presented with numbness, cold sensation, and pain in the left arm. Contrast-enhanced computed tomography (CT) revealed occlusion of the left brachial artery as well as a pedunculated mass inside the proximal ascending aorta (**Fig. 1**). Embolectomy was performed, and ischemic symptoms of the arm then resolved. Histopathology revealed the emboli to be thrombi. For differential diagnosis of the intra-aortic mass, positron emission tomography (PET)–CT was performed, which did not show any uptake of ^18^F-fluorodeoxyglucose (FDG) in the mass. The results of laboratory screening for protein C, protein S, and antithrombin III deficiencies were deemed to be negative. Serology revealed a slightly elevated lupus anticoagulant level at 1.39 (normal <1.30). Anticardiolipin and anti-β2 glycoprotein-I antibodies were negative.

**Figure 2:**
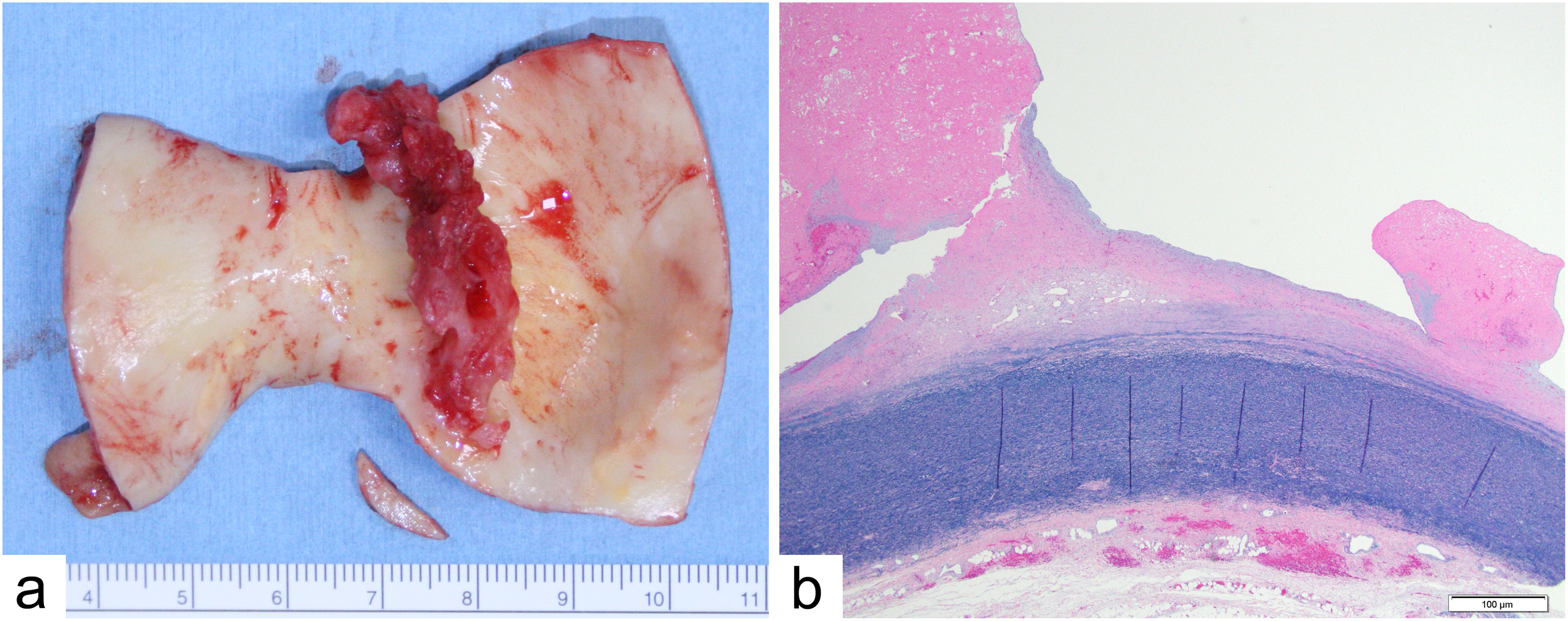
Fig. 2 (**a**) Macroscopic and (**b**) microscopic views of the resected specimen. Victoria blue-hematoxylin and eosin staining. Original magnification ×20.

**Figure 3:**
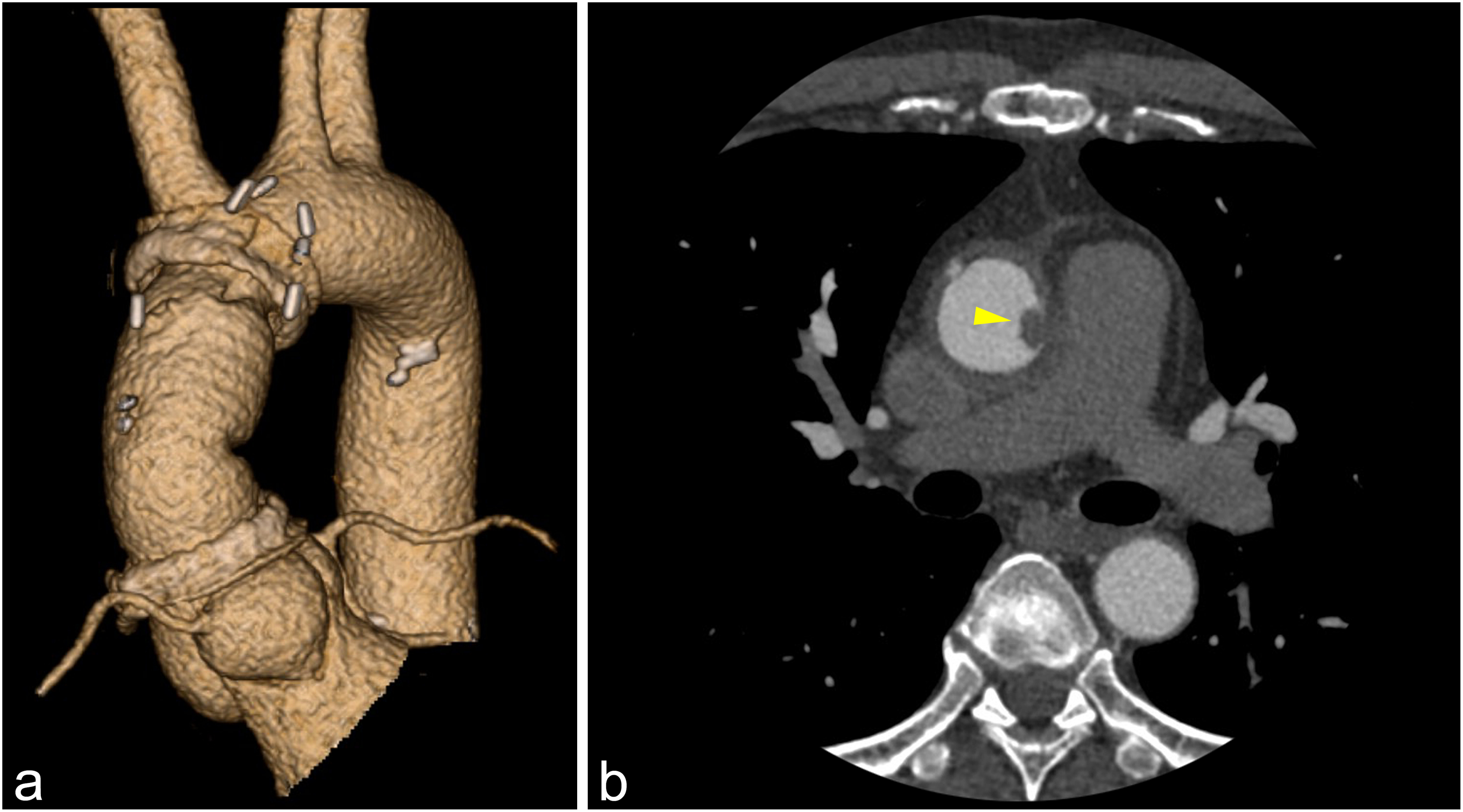
Fig. 3 Postoperative computed tomography scans at (**a**) 1 week and (**b**) 6 months after surgery. The arrowhead indicates a thrombus formed inside the graft.

The ascending aorta was replaced under circulatory arrest and selective cerebral perfusion 13 days after the first operation. On opening the aorta, gelatinous mass measuring 40 mm×10 mm was recognized at the sinotubular junction above the right coronary sinus. Neither an atherosclerotic lesion nor thickening of the aortic wall was recognized at the site of attachment. To obtain a 5-mm surgical margin, incision was extended into the right coronary sinus, and the mass was resected en bloc with the aortic wall (**Fig. 2a**, Video). The ascending aorta was then replaced with a 24-mm Triplex vascular prosthesis (Terumo Corporation, Tokyo, Japan) (**Fig. 3a**).

Histopathology revealed the mass to be a thrombus (**Fig. 2b**). No malignant cells were recognized at the site of attachment on the aortic wall. Therefore, an anticoagulation therapy with warfarin was initiated. The postoperative course was noted to be uneventful, and the patient was discharged on day 10 after surgery. Since the patient was a construction site worker and was frequently exposed to bruises, anticoagulation therapy was ceased 3 months after the surgery as per the patient’s request. As the second serological test again detected lupus anticoagulant (1.46), the diagnosis of APS was confirmed. Anticardiolipin and anti-β2 glycoprotein-I antibodies were not detected. The patient did not agree to resume taking warfarin.

Contrast-enhanced CT performed 6 months after surgery revealed a thrombus measuring 8 mm×5 mm inside the graft (**Fig. 3b**). Despite our persuasion, the patient refused to take warfarin. Alternatively, edoxaban (60 mg/day) and aspirin (100 mg/day) were initiated. The resolution of the thrombus was confirmed via CT 4 months later. The patient has been relapse-free for 2 years while continuing to take the drugs.

## Discussion

APS has been characterized by the development of arterial and venous thromboses at various sites. However, thrombosis of the aorta is extremely rare. Cases involving the abdominal aorta^1)^ and distal aortic arch^2)^ were reported anecdotally. Two cases of mural thrombosis in the ascending aorta have been reported previously, and both of these patients showed concomitant right ventricular infarction.^3,4)^

According to the diagnostic criteria for APS,^5)^ lupus anticoagulant, anticardiolipin, or anti-β2 glycoprotein-I must be positive on two occasions, not less than 12 weeks apart. Theoretically, it is impossible to confirm the diagnosis of APS in the initial presentation of a patient with acute embolism or thrombosis necessitating urgent surgery. APS should be suspected when a screening test detects one of the abovementioned antibodies. However, this does not indicate that the mass is a thrombus because aortic involvement in APS patients is a rare manifestation.

It remains difficult to distinguish between an aortic mural thrombus and an aortic tumor due to similar CT findings and clinical presentation. In a study by Kato et al.,^6)^ a case of protruding angiosarcoma of the aortic arch was reported. The patient presented with arterial embolism of the left upper limb. ^18^F-FDG PET–CT appears to have been of some help in clarifying the diagnosis. In the case of Kato et al.,^6)^ a minor increase in the uptake of ^18^F-FDG (maximum standard uptake value 1.193) was found at the location corresponding to the mass. Yokawa et al.^7)^ reported that PET–CT identified the metastatic lesions of angiosarcoma of the descending aorta. However, the sensitivity and specificity of PET–CT are unknown, owing to the rarity of the disease.

The treatment of an aortic mural thrombus is usually a straight choice between graft replacement and simple thrombectomy.^3,4,8)^ However, the difficulty of differentiating a thrombus from a malignant tumor preoperatively makes the procedural strategy complicated. Moreover, application of an intraoperative rapid pathological diagnosis is not straightforward because both procedures, i.e., graft replacement and simple thrombectomy, require circulatory arrest. At our institution, it takes approximately 30 min to diagnose thrombi using frozen sections stained with hematoxylin and eosin, including the time for specimen transport. It is possible to remove the mass first under short circulatory arrest and then perform graft replacement, applying second circulatory arrest in case the pathological examination turns out to be malignant.

Although replacement of the ascending aorta for acute aortic dissection in a patient with APS has been reported,^9)^ incidence of thrombosis after graft replacement remains to be poorly understood. To the best of our knowledge, this is the first report of thrombosis following replacement of the aorta with a vascular prosthesis in APS. Although warfarin was administered postoperatively, it was discontinued as per the patient’s request. Even after the diagnosis of APS was confirmed, the patient refused to take warfarin and this led up to the recurrence of thrombosis in the graft. Since the patient refused taking warfarin, edoxaban was alternatively administered in conjunction with aspirin. Consequently, the thrombus resolved, and there was no relapse since the patient continued taking the drugs. However, all the major guidelines recommend vitamin K antagonists in patients with arterial thrombotic events.^10)^ Although some guidelines^11–13)^ accept the use of direct oral anticoagulants in patients with venous thrombotic events and single/double antiphospholipid antibody positivity, vitamin K antagonists remain the first line of anticoagulants in APS.

In retrospect, surgical strategy using simple thrombectomy might be an optimal choice due to the following reasons: (1) no uptake of ^18^F-FDG in the mass was confirmed through PET–CT; (2) positive lupus anticoagulant indicated the possibility of APS; and (3) thrombosis following graft replacement in patients with APS was uncertain.

## Conclusion

To the best of our knowledge, this is the first report on graft thrombosis in APS. Following aortic replacement, seamless and long-term anticoagulation with vitamin K antagonists is strongly recommended.

## Supplementary Information

Supplementary movie is available at the online article sites on J-STAGE and PMC.
